# Association Between Obesity and Short-And Long-Term Mortality in Patients With Acute Respiratory Distress Syndrome Based on the Berlin Definition

**DOI:** 10.3389/fendo.2020.611435

**Published:** 2021-02-12

**Authors:** Wei Zhang, Yadan Wang, Weijie Li, Jun Wang

**Affiliations:** ^1^Department of Respiratory and Critical Care Medicine, Shaanxi Provincial People’s Hospital, Xi’an, China; ^2^Medical Department, Ruibiao (Wuhan) Biotechnology Co. Ltd, Wuhan, China

**Keywords:** ARDS, obesity, body mass index, 28-day mortality, 1-year mortality

## Abstract

**Purpose:**

Acute respiratory distress syndrome (ARDS) is one of the most common causes of death in intensive care units (ICU). Previous studies have reported the potential protective effect of obesity on ARDS patients. However, these findings are inconsistent, in which less was reported on long-term prognosis and diagnosed ARDS by Berlin definition. This study aimed to investigate the relationship between obesity and short-term and long-term mortality in patients with ARDS based on the Berlin Definition.

**Methods:**

This is a retrospective cohort study from the Medical Information Mart for Intensive Care III (MIMIC-III) database, in which all the patients were diagnosed with ARDS according to the Berlin definition. The patients were divided into four groups according to the WHO body mass index (BMI) categories. The multivariable logistic regression and Cox regression analysis were used to investigate the relationship between BMI and short-term and long-term mortality.

**Result:**

A total of 2,378 patients with ARDS were enrolled in our study. In-hospital mortality was 27.92%, and 1,036 (43.57%) patients had died after 1-year follow-up. After adjusting for confounders, the in-hospital and 1-year mortality risks of obese patients were significantly lower than those of normal weight (OR 0.72, 95%CI 0.55–0.94, P=0.0168; HR 0.80, 95%CI 0.68–0.94 P=0.0084; respectively), while those mortality risks of underweight patients were higher than normal weight patients (P=0.0102, P=0.0184; respectively). The smooth curve showed that BMI, which was used as a continuous variable, was negatively correlated with in-hospital and 1-year mortality. The results were consistent after being stratified by age, gender, race, type of admission, severity of organ dysfunction, and severity of ARDS. The Kaplan-Meier survival curves showed that obese patients had significant lower 1-year mortality than normal weight patients.

**Conclusion:**

We found that obesity was associated with decreased risk of short-term and long-term mortality in patients with ARDS.

## Introduction

Acute respiratory distress syndrome (ARDS) is a fatal form of acute respiratory failure, which is caused by direct (pneumonia or aspiration) or indirect lung injuries (sepsis or trauma) (1). It is one of the most common causes of death in intensive care units (ICU) and an important public health problem (2). In a recent study which involved 29,114 patients in the ICU from 50 countries, 10% of the patients who were admitted to ICU and 23% of the patients on mechanical ventilation were diagnosed with ARDS. Approximately 35–46% of patients with ARDS died consequently during hospitalization (3). Despite the efforts in early diagnosis and treatment, to our knowledge, there is no available treatment which can aim directly at the pathological mechanism of ARDS, and mechanical ventilation and supportive care are still the main approaches (1, 2, 4). The mortality of ARDS is still very high. Therefore, the recognition of poor prognostic factors is helpful for clinicians to adjust treatment strategies early and ultimately improve the prognosis of patients with ARDS.

Obesity has been on the rise over the past 30 years due to the changes of our feeding habits and life Style. According to the 2017 Global Nutrition Report, 2 billion adults were obese or overweight worldwide and about one in five patients in ICU was obese (5). In recent years, some studies have shown that obesity can reduce mortality in critically ill patients (5–7), such as those with chronic heart disease, chronic renal insufficiency, and sepsis, this phenomenon is termed obesity paradox (8–11); Yet whether this phenomenon exists in ARDS is still controversial (12–20). Most of the previous studies were conducted with a limited sample size, and ARDS as defined by the Berlin standard was rarely applied. Moreover, these studies mainly focused on short-term prognosis, the long-term prognosis is unclear. Our study investigated a cohort of patients with ARDS based on the current definitions and guidelines (Berlin definition), with the primary outcomes of in-hospital and 1-year mortality, to assess the effect of obesity on mortality and the course of disease.

## Methods and Materials

### Data Sources

The present study is a retrospective cohort study of 2,378 patients with ARDS according to the Berlin definition. All the data used for analysis were obtained from the Medical Information Mart for Intensive Care III (MIMIC-III), which is a single-center and freely accessible database and contains 38,597 adult patients (aged over 16) during 2001 to 2012 in the ICU of the Beth Israel Deaconess Medical Center in Boston. We were obliged to complete the online course and pass the online exams (no. 6182750) to gain access to the database. The establishment of the MIMIC III database was approved by the institutional review board of Beth Israel deacons Medical Center and Massachusetts Institute of Technology. Because all the data of health information are anonymous, the informed consent was not required.

### Patients

The MIMIC-III database contains the admission and discharge diagnosis information of all patients. Combined with the diagnostic information recorded in the database and the Berlin standard, we selected the patients diagnosed as ARDS for analysis. Our inclusion criteria were as follows: (1) Patients were older than 16 years and stayed in the ICU more than 48 h; (2) Patients had an arterial oxygen partial pressure (PaO2)/fraction of inspired oxygen (FiO2) < 300 mmHg and positive end-expiratory pressure (PEEP) ≥ 5cm H_2_O on the first day of ICU admission; (3) Patients with acute onset were assumed to have recently used mechanical ventilation during ICU stay; (4) Patients whose chest radiographs showed bilateral infiltration, and absence of heart failure. Patients lacking of height or weight were excluded. If a patient was admitted repeatedly during the study period, we used only the record of his/her first ICU admission.

The patients’ BMI was calculated as weight (Kg)/height^2^ (m). According to the BMI international classification of World Health Organization(WHO), we divided the study population into four groups: underweight (BMI < 18.5 kg/m^2^), normal weight (BMI ≥ 18.5, < 25 kg/m^2^), overweight (BMI ≥ 25, < 30 kg/m^2^) and obese (BMI ≥ 30 kg/m^2^).

According to the Berlin Definition, ARDS were classified into mild (> 200 mmHg, ≤ 300 mmHg), moderate (> 100 mmHg, ≤ 200 mmHg), and severe (< 100 mmHg) based on the PaO2/FiO2 ratio.

### Variables and Outcome Measures

We used the Structured Query Language to extract the data and the cords of the Structured Query Language were obtained from https://github.com/MIT-LCP/mimic-website. We extracted or calculated the following variables, including the baseline characteristics (age, gender, ethnicity, admission type, ICU type), the patients’ comorbidity, the vital signs within 24h after ICU admission (heart rate, temperature, mean arterial pressure and SPO_2_), severity of organ dysfunction (Simplified Acute Physiology Score, SAPSII; Oxford Acute Severity of Illness Score, OASIS; Sequential Organ Failure Assessment, SOFA) and the characteristics of mechanical ventilation on the first day. If a variable was measured multiple times during the study period, we took its average value. The serious scores of organ dysfunction was estimated for all patients within 24 h of ICU admission.

The primary outcomes were the in-hospital and 1-year mortality following ICU admission, and the secondary outcomes were ICU mortality, ICU length of stay, and in-hospital length of stay.

### Statistical Analysis

We used the EmpowerStats software (www.empowerstats.com version R.3.4.3) and statistical software package R to process and analyze all our data. The categorical variables were presented as percentages and the continuous variables were expressed as the mean (SD) or IQR in the tables in our study. All the variables were compared by student t-test (normal distribution) or Mann-Whitney (non-normal distribution) when they were categorical variables, and by Kruskal-Wallis test when they were continuous variables. A two-tailed P value < 0.05 was considered statistically significant.

Multivariable logistic regression analysis and smooth curve fitting were performed to test the independent effects of obesity on ICU and in-hospital mortality with crude and full models. Multivariable Cox regression models and generalized linear models with a logit link were used to test the independent effects of obesity on 1-year all-cause mortality. The adjusting variables included age, gender, ethnicity, admission type, ICU type, sepsis, chronic pulmonary disease, renal failure, liver disease, metastatic cancer, chronic heart disease, cerebrovascular disease, Elixhauser comorbidity score, vital signs within 24h after ICU admission, arterial pH, lactic acid and the support treatment. Additionally, the log-rank test in the Kaplan-Meter survival analysis was used to compare the different survival rates between each group. Furthermore, a subgroup analysis was performed to determine whether there were differences among each subgroup in BMI prediction of clinical outcomes.

## Results

### Characteristics and Outcomes of Patients

**Figure 1:**
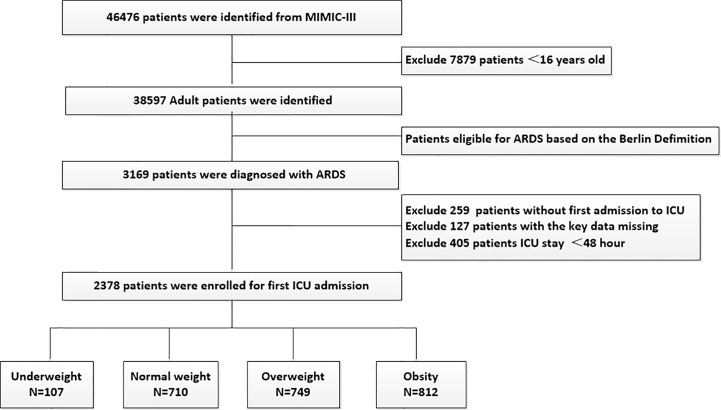
Flow chart of the current study.

The flowchart of study cohort selection was shown in **Figure 1**.** **A total of 2,378 patients with ARDS were included in this study, containing 107 underweight patients, 710 normal weight patients, 749 overweight patients, and 812 obese patients. **Table 1** displayed the characteristics of the study participants and **Table 2** displayed the clinical outcomes of the subjects across the BMI strata.

**Table 1:** Characteristics of the study patients according to BMI.

Variables	All patients	Underweight <18.5	Normal weight ≥18.5, <25	Overweight ≥25, <30	Obesity ≥30	P-value
Baseline characteristics						
n	2378	107	710	749	812	
Age (years)	61.90 ± 17.48	67.22 ± 16.72	64.10 ± 19.22	62.09 ± 17.58	59.12 ± 15.33	<0.001
Male	1425 (59.92%)	60 (56.07%)	442 (62.25%)	488 (65.15%)	435 (53.57%)	<0.001
Ethnicity						
Caucasian	1647 (69.26%)	69 (64.49%)	482 (67.89%)	525 (70.09%)	571 (70.32%)	0.200
Black	522 (21.95%)	27 (25.23%)	161 (22.68%)	173 (23.10%)	161 (19.83%)	
Others	209 (8.79%)	11 (10.28%)	67 (9.44%)	51 (6.81%)	80 (9.85%)	
Admission type						
Elective	335 (14.09%)	13 (12.15%)	96 (13.52%)	96 (12.82%)	130 (16.01%)	0.262
Emergency/Urgent	2043 (85.91%)	94 (87.85%)	614 (86.48%)	653 (87.18%)	682 (83.99%)	
ICU type						
CCU/CSRU	236 (9.92%)	8 (7.48%)	59 (8.31%)	79 (10.55%)	90 (11.08%)	0.059
MICU	1251 (52.61%)	68 (63.55%)	367 (51.69%)	379 (50.60%)	437 (53.82%)	
SICU/TSICU	891 (37.47%)	31 (28.97%)	284 (40.00%)	291 (38.85%)	285 (35.10%)	
Comorbidity						
Chronic pulmonary disease	624 (26.24%)	38 (35.51%)	176 (24.79%)	167 (22.30%)	243 (29.93%)	<0.001
Renal failure	129 (5.42%)	12 (11.21%)	97 (13.66%)	124 (16.56%)	112 (13.79%)	0.240
Liver disease	252 (10.60%)	11 (10.28%)	61 (8.59%)	86 (11.48%)	94 (11.58%)	0.215
Metastatic cancer	156 (6.56%	11 (10.28%)	54 (7.61%)	50 (6.68%)	41 (5.05%)	<0.081
Chronic heart disease	927 (38.98%)	40 (37.38%)	301 (42.39%)	271 (36.18%)	315 (38.79%)	0.108
Cerebrovascular disease	276 (11.61%)	14 (13.08%)	77 (10.85%)	90 (12.02%)	95 (11.70%)	0.859
Diabetes	626 (26.32%)	13 (12.15%)	127 (17.89%)	179 (23.90%)	307 (37.81%)	<0.001
Sepsis	1322 (55.59%)	60 (56.07%)	381 (53.66%)	412 (55.01%)	469 (57.76%)	0.435
Elixhauser comorbidity score	8.50 ± 7.85	10.76 ± 8.74	8.73 ± 7.76	8.56 ± 7.70	7.95 ± 7.90	0.004
Vital signs within 24h after ICU admission						
Heart rate (bpm)	90.89 ± 16.80	91.65 ± 17.09	91.21 ± 16.78	90.68 ± 16.16	90.70 ± 17.37	0.874
MAP(mmHg)	76.16 ± 9.94	73.67 ± 8.90	76.00 ± 10.15	75.44 ± 9.49	77.30 ± 10.17	<0.001
Temperature(℃)	37.08 ± 0.77	36.75 ± 0.77	37.02 ± 0.76	37.10 ± 0.76	37.15 ± 0.78	<0.001
SPO_2_	96.81 ± 3.85	96.22 ± 6.59	96.96 ± 3.17	96.80 ± 4.17	96.76 ± 3.58	0.287
Severity of organ dysfunction						
SAPSII	44.51 ± 14.76	46.78 ± 14.38	45.47 ± 14.31	44.56 ± 14.76	43.31 ± 15.10	0.012
OASIS	38.05 ± 8.26	39.73 ± 8.17	38.45 ± 7.87	37.95 ± 8.44	37.56 ± 8.39	0.028
SOFA	7.03 ± 3.55	6.35 ± 3.46	6.92 ± 3.56	7.05 ± 3.45	7.21 ± 3.64	0.077
Laboratory data on the first day after ICU admission						
Arterial pH	7.36 ± 0.08	7.34 ± 0.09	7.36 ± 0.08	7.36 ± 0.07	7.35 ± 0.08	0.123
PaCO2(mm Hg)	42.40 ± 9.70	42.50 ± 10.06	41.58 ± 9.91	41.67 ± 8.73	43.80 ± 10.18	<0.001
Lactic acid	2.00 ± 2.20	1.61 ± 1.80	1.95 ± 2.16	2.15 ± 2.43	1.94 ± 2.05	0.057
PaO2/FiO2 (mmHg)	142.50 ± 65.73	160.80 ± 70.42	142.87± 67.77	140.13± 64.08	141.95± 64.50	0.025
Need of support in the first 24 h						
Renal replacement therapy	129 (5.42%)	4 (3.74%)	43 (6.06%)	38 (5.07%)	44 (5.42%)	0.723
Vasopressor	1212 (50.97%)	51 (47.66%)	357 (50.28%)	391 (52.20%)	413 (50.86%)	0.786
Characteristics of mechanical ventilation on the first day after ICU admission						
Tidal volume (ml/kg PBW)	9.08 ± 3.74	8.34 ± 3.71	8.62 ± 2.30	8.92 ± 2.23	9.73 ± 5.41	<0.001
PEEP (cmH2O)	7.44 ± 3.36	6.32 ± 2.52	7.16 ± 2.98	7.30 ± 3.30	7.95 ± 3.73	<0.001
Plateau pressure (cmH2O)	22.66 ± 6.02	20.23 ± 5.89	21.80 ± 6.43	22.55 ± 5.73	23.82 ± 5.69	<0.001
Minute ventilation (l/min)	9.10 ± 5.72	8.34 ± 7.67	8.82 ± 6.71	8.98 ± 3.72	9.56 ± 5.98	0.027
FiO2 (%)	60.54 ± 12.67	56.95 ± 14.64	59.68 ± 12.30	60.82 ± 12.45	61.49 ± 12.81	<0.001

BMI, Body Mass Index; ICU, intensive care units; CCU, coronary care unit; CSRU, cardiac surgery recovery unit; MICU, medical intensive care unit; SICU, surgical intensive care unit; TSICU, thoracic surgery Intensive care unit; MAP, Mean Arterial Pressure; PaO2/FiO2, arterial oxygen partial pressure/fraction of inspired oxygen; SAPSII, Simplified Acute Physiology Score; OASIS, Oxford Acute Severity of Illness Score; SOFA, Sequential Organ Failure Assessment; PBW, predicted body weight; PEEP, positive end-expiratory pressure; bpm, breaths per minute.

**Table 2:** Outcomes of subjects across the BMI strata.

Variables	All patients	underweight < 18.5	Normal weight ≥ 18.5, <25	Overweight ≥ 25, <30	Obesity ≥ 30	P-value
n	2378	107	710	749	812	
Time in ICU (days)	13.10 ± 12.14	11.48 ± 10.09	12.5 ± 12.45	13.7 ± 12.78	13.2 ± 11.45	0.124
Time in hospital (days)	22.58 ± 18.83	21.00 ± 14.23	21.64 ± 17.75	23.35 ± 20.35	22.89 ± 18.80	0.261
ICU mortality	556 (23.38%)	42 (39.25%)	185 (26.06%)	184 (24.57%)	145 (17.86%)	<0.001
In-hospital mortality	664 (27.92%)	50 (46.73%)	217 (30.56%)	221 (29.51%)	176 (21.67%)	<0.001
1-year mortality	1036 (43.57%)	72 (67.29%)	351 (49.44%)	332 (44.33%)	281 (34.61%)	<0.001

The average age of the study participants was 61.90 ± 17.48 years and 59.92% of participants were male. In total, 664 participants (27.92%) and 1,036 participants (43.57%) died in hospital and within one year, respectively. Obese patients tended to be younger and obese female patients were more than normal weight patients. In obese patients, the incidence of common comorbidities such as diabetes and chronic lung disease is significantly higher. The SAPSII and OASIS scores within 24-hour of ICU admission were significantly lower in obese patients than those in normal weight patients. These imbalanced variables may be confounding and were adjusted in subsequent analyses.

Furthermore, obese patients needed higher PEEP, plateau pressure, minute ventilation, and FiO2 than normal weight patients. And they had a lower ICU in-hospital and 1-year mortality than normal weight patients. There was no difference on ICU length of stay and in-hospital length of stay between obese patients and normal weight patients.

### Obesity and Short-Term Mortality

The univariate logistic regression analysis for ICU and in-hospital mortality was shown in **Table S1**. There was a significant association between patients’ variables (age, ethnicity, BMI, admission type, ICU type, renal failure, liver disease, metastatic cancer, chronic heart disease, sepsis, vital signs within 24h after ICU admission, arterial pH, lactic acid, SAPSII, OASIS, SOFA, plateau pressure and FiO2) and in-hospital mortality.

In multivariable logistic regression analysis, after adjusting for the clinical confounders listed, we found that obese patients had significant lower risks of the in-hospital (OR 0.72, 95%CI 0.55–0.94, P=0.0168) and ICU (OR 0.83, 95%CI 0.53–0.93 P=0.0140) mortality than patients with normal weights, while the underweight patients had significant higher risks of the in-hospital (OR 1.87 95%CI 1.16, 3.02 P=0.0102) and ICU (OR 1.76 95%CI 1.08, 2.87 P=0.0244 **Table 3**) mortality.

**Table 3:** Multivariate logistics regression analysis for ICU and in-hospital mortality in BMI groups.

BMI group	Non-adjusted	Adjust I	Adjust II
ICU mortality	OR 95% CI p-value	OR 95% CI p-value	OR 95% CI p-value
Normal weight	1.0 (reference)	1.0 (reference)	1.0 (reference)
Underweight	1.83 (1.20, 2.80) 0.0049	1.73 (1.12, 2.66) 0.0135	1.76 (1.08, 2.87) 0.0244
Overweight	0.92 (0.73, 1.17) 0.5128	0.97 (0.76, 1.23) 0.7925	0.93 (0.71, 1.22) 0.5952
Obesity	0.62 (0.48, 0.79) 0.0001	0.70 (0.54, 0.90) 0.0049	0.70 (0.53, 0.93) 0.0140
p for trend	<0.0001	0.0047	0.0145
In-hospital mortality	OR 95% CI p-value	OR 95% CI p-value	OR 95% CI p-value
Normal weight	1.0 (reference)	1.0 (reference)	1.0 (reference)
Underweight	1.99 (1.32, 3.01) 0.0010	1.89 (1.24, 2.88) 0.0032	1.87 (1.16, 3.02) 0.0102
Overweight	0.95 (0.76, 1.19) 0.6596	0.99 (0.79, 1.25) 0.9590	0.97 (0.75, 1.25) 0.7866
Obesity	0.63 (0.50, 0.79) <0.0001	0.71 (0.56, 0.90) 0.0052	0.72 (0.55, 0.94) 0.0168
p for trend	<0.0001	0.0048	0.0186

Adjusted I for age, gender and ethnicity;

Adjusted II for age, gender, ethnicity, admission type, ICU type, sepsis, chronic pulmonary disease, renal failure, liver disease, metastatic cancer, chronic heart disease, cerebrovascular disease, Elixhauser comorbidity score, vital signs within 24h after ICU admission, SAPSII, SOFA, OASIS, arterial pH, lactic acid, renal replacement therapy and vasopressor.

**Figure 2:**
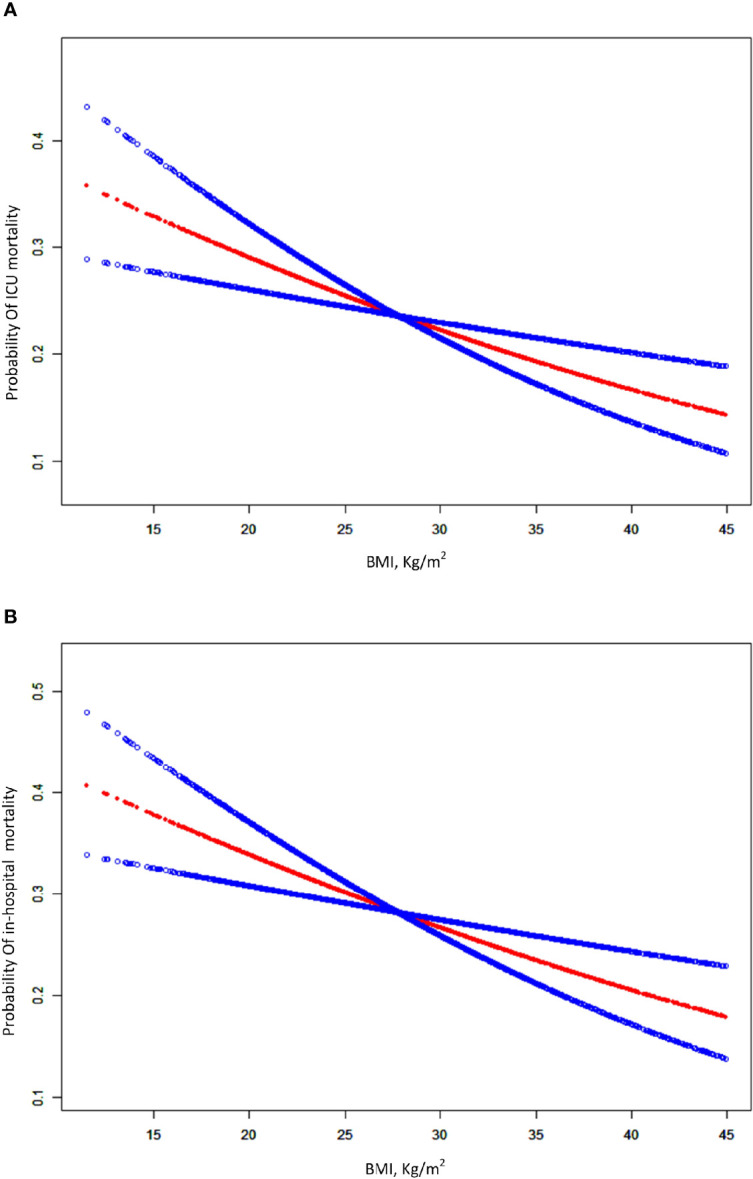
A smooth curve fitting for the relationship between BMI and the risk of ICU and in-hospital mortality. **(A)** Association between BMI and ICU mortality for patients with ARDS. **(B)** association between BMI and in-hospital for patients with ARDS. The resulting figures show the risk of mortality in the y-axis and the BMI (continuous variable) in the x-axis. A negative relationship between BMI and the risk of short-term mortality was observed after adjusting for age, gender, ethnicity, admission type, ICU type, sepsis, chronic pulmonary disease, renal failure, liver disease, metastatic cancer, chronic heart disease, cerebrovascular disease, Elixhauser comorbidity score, vital signs within 24h after ICU admission, SAPSII, SOFA, OASIS, arterial pH, lactic acid, renal replacement therapy and vasopressor by spline smoothing plot.

Taking BMI as a continuous variable, we used the adjusted spline smoothing fitting to visually assess the relationship between BMI and in-hospital mortality, which were presented in **Figure 2**. The results showed that BMI was negatively associated with in-hospital mortality in crude and adjusted models.

### Obesity and Long-Term Mortality

**Figure 3:**
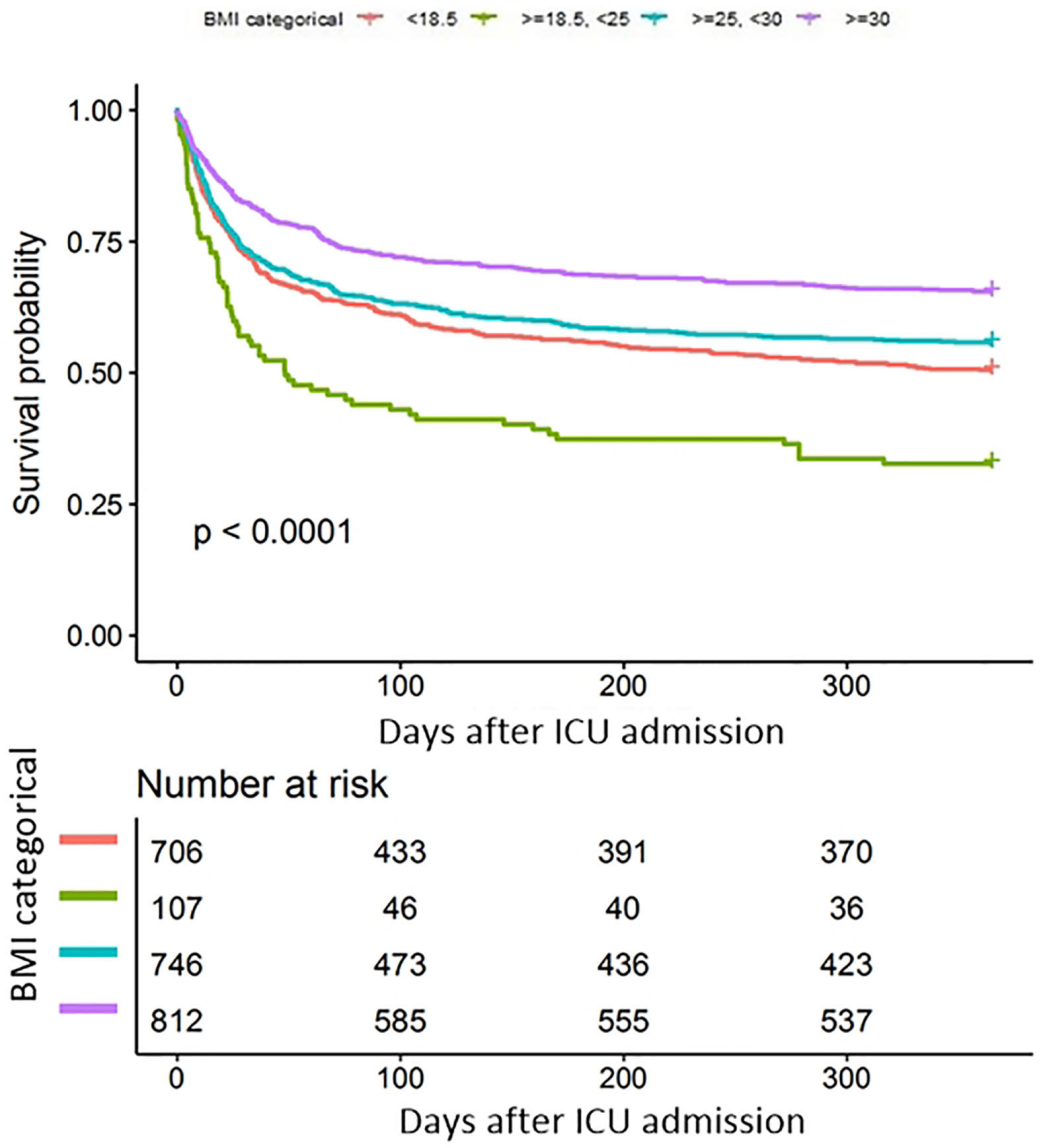
Kaplan-Meier (K-M) survival curves of 1-year mortalities by BMI categorical.

**Figure 4:**
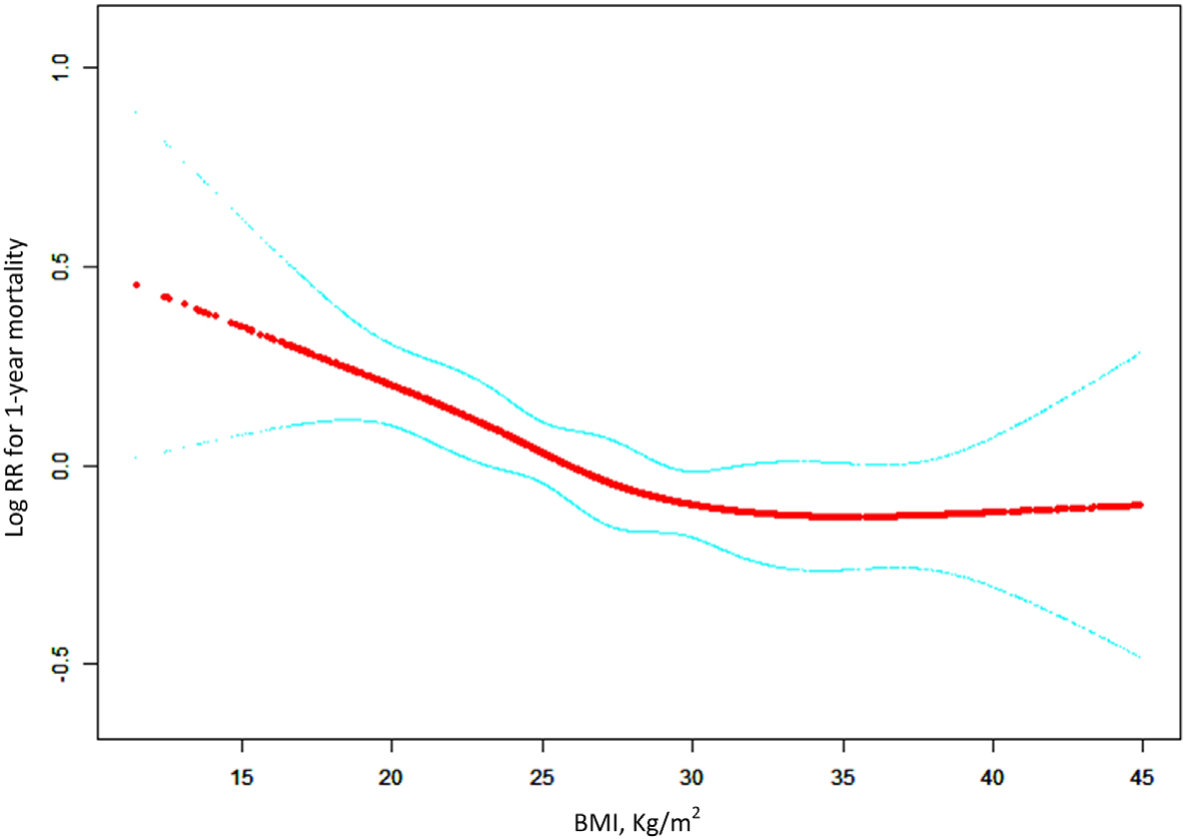
General additive models demonstrate the relationship between BMI and the risk of 1-year mortality in ARDS patients. The resulting figures show the predicted log(relative risk) in the y-axis and the BMI in the x-axis. The model was adjusted for age, gender, ethnicity, admission type, ICU type, sepsis, chronic pulmonary disease, renal failure, liver disease, metastatic cancer, chronic heart disease, cerebrovascular disease, Elixhauser comorbidity score, vital signs within 24h after ICU admission, SAPSII, SOFA, OASIS, arterial pH, lactic acid, renal replacement therapy and vasopressor.

In univariate COX analysis (**Table S2**), BMI showed a strong relation with long-term survival as illustrated by the Kaplan–Meier curves (log-rank test P < 0.001, **Figure 3**). In the multivariable Cox regression analysis, obese patients had significant lower risks of 1-year mortality (HR 0.63, 95%CI 0.53–0.73 P<0.0001) than patients with normal weights. After adjusting for potential confounders, the association remained independent (HR 0.80, 95%CI 0.68–0.94 P=0.0084, **Table 4**, model 2). Moreover we used the generalized additive models to visually assess the relationship between BMI and 1-year mortality (**Figure 4**). The result was similar with the short-term outcomes.

**Table 4:** Multivariable Cox regression analysis for 1-year mortality in BMI groups.

BMI group	Non-adjusted	Adjust I	Adjust II
1-year mortality	HR 95% CI p-value	HR 95% CI p-value	HR 95% CI p-value
Normal weight	1.0 (reference)	1.0 (reference)	1.0 (reference)
Underweight	1.66 (1.29, 2.14) <0.0001	1.54 (1.20, 1.99) 0.0008	1.37 (1.05, 1.78) 0.0184
Overweight	0.87 (0.75, 1.01) 0.0765	0.93 (0.80, 1.09) 0.3683	0.92 (0.79, 1.08) 0.3101
Obesity	0.63 (0.53, 0.73) <0.0001	0.73 (0.63, 0.86) 0.0002	0.80 (0.68, 0.94) 0.0084
p for trend	<0.0001	<0.0001	0.0061

Adjusted I for age, gender and ethnicity;

Adjusted II for age, gender, ethnicity, admission type, ICU type, sepsis, chronic pulmonary disease, renal failure, liver disease, metastatic cancer, chronic heart disease, cerebrovascular disease, Elixhauser comorbidity score, vital signs within 24h after ICU admission, SAPSII, SOFA, OASIS, arterial pH, lactic acid, renal replacement therapy and vasopressor

### Subgroup Analyses

The subgroup analyses for the relationship between BMI and the in-hospital and 1-year mortality were presented in **Tables 5** and **6**, which were performed according to age, gender, race, type of admission, severity of ARDS, SAPSII, OASIS and SOFA scores. The results showed that in different subgroups, the relationship between BMI and the risk of in-hospital and 1-year mortality stably existed after careful adjustments. The association between obesity and long-term mortality was only not observed in other race patients (OR 1.03, 95% CI 0.53–1.99, P = 0.9269).

**Table 5:** Subgroup analysis for the effect of obesity on risk of in-hospital mortality after adjusting for confounding factors.

BMI	N	Normal weight reference	Underweight OR 95% CI p-value	Overweight OR 95%CI p-value	Obesity OR 95% CI p-value
Age					
<65	1266	1.0	3.44 (1.59, 7.45) 0.0018	0.91 (0.60, 1.38) 0.6572	0.83 (0.55, 1.25) 0.3771
≥65	1112	1.0	1.47 (0.75, 2.89) 0.2617	0.99 (0.70, 1.40) 0.9518	0.62 (0.41, 0.92) 0.0192
Gender					
Male	1425	1.0	1.88 (0.93, 3.81) 0.0781	0.95 (0.68, 1.34) 0.7866	0.67 (0.46, 0.98) 0.0368
Female	953	1.0	2.48 (1.16, 5.32) 0.0197	0.99 (0.63, 1.54) 0.9574	0.80 (0.51, 1.25) 0.3309
Ethnicity					
Caucasian	1647	1.0	2.25 (1.18, 4.28) 0.0132	0.79 (0.55, 1.12) 0.1802	0.59 (0.40, 0.86) 0.0061
black	522	1.0	0.72 (0.24, 2.17) 0.5588	1.09 (0.64, 1.85) 0.7634	0.73 (0.40, 1.31) 0.2931
other	209	1.0	3.45 (0.49, 24.45) 0.2148	1.26 (0.31, 5.01) 0.7470	0.94 (0.25, 3.57) 0.9254
Admission type					
Elective	335	1.0	2.73 (0.46, 16.15) 0.2672	0.90 (0.37, 2.21) 0.8230	0.84 (0.34, 2.10) 0.7163
Emergency/Urgent	2043	1.0	1.69 (1.01, 2.82) 0.0468	0.93 (0.70, 1.24) 0.6375	0.66 (0.48, 0.89) 0.0072
SAPSII					
<43	1151	1.0	1.65 (0.67, 4.06) 0.2743	1.35 (0.87, 2.08) 0.1817	0.51 (0.31, 0.85) 0.0103
≥43	1227	1.0	2.18 (1.15, 4.10) 0.0162	0.82 (0.59, 1.15) 0.2532	0.90 (0.63, 1.27) 0.5380
SOFA					
<7	1173	1.0	1.65 (0.84, 3.25) 0.1442	0.91 (0.61, 1.36) 0.6567	0.52 (0.33, 0.83) 0.0055
≥7	1205	1.0	2.73 (1.22, 6.12) 0.0145	1.02 (0.71, 1.47) 0.9172	0.91 (0.63, 1.31) 0.6021
OASIS					
<38	1161	1.0	3.05 (1.33, 7.00) 0.0086	1.12 (0.74, 1.69) 0.5947	0.58 (0.36, 0.94) 0.0271
≥38	1217	1.0	1.64 (0.87, 3.08) 0.1239	0.88 (0.62, 1.25) 0.4692	0.86 (0.60, 1.23) 0.4162
ARDS					
Mild	493	1.0	1.02 (0.85, 1.51) 0.2080	1.05 (0.57, 1.93) 0.8778	0.65 (0.34, 1.22) 0.1781
Moderate	1110	1.0	2.75 (1.34, 5.65) 0.0060	1.04 (0.70, 1.55) 0.8423	0.72 (0.46, 1.10) 0.1313
Severe	775	1.0	2.03 (0.81, 5.05) 0.1297	0.87 (0.57, 1.33) 0.5068	0.79 (0.51, 1.23) 0.2973

**Table 6:** Subgroup analysis for the effect of obesity on risk of 1-year mortality after adjusting for confounding factors.

BMI	N	Normal weight reference	Underweight HR 95% CI p-value	Overweight HR 95%CI p-value	Obesity HR 95% CI p-value
Age					
<65	1266	1.0	2.09 (1.36, 3.22) 0.0007	0.94 (0.72, 1.22) 0.6283	0.85 (0.65, 1.10) 0.2182
≥65	1112	1.0	1.26 (0.90, 1.76) 0.1817	0.88 (0.73, 1.07) 0.1966	0.74 (0.59, 0.92) 0.0069
Gender					
Male	1425	1.0	1.50 (1.05, 2.16) 0.0272	0.89 (0.73, 1.09) 0.2565	0.78 (0.62, 0.98) 0.0291
Female	953	1.0	1.24 (0.83, 1.85) 0.3006	0.95 (0.73, 1.22) 0.6696	0.82 (0.63, 1.05) 0.1179
Ethnicity					
Caucasian	1647	1.0	1.47 (1.05, 2.04) 0.0239	0.84 (0.70, 1.02) 0.0832	0.73 (0.60, 0.90) 0.0025
black	522	1.0	1.05 (0.61, 1.82) 0.8543	1.02 (0.76, 1.38) 0.8914	0.86 (0.61, 1.22) 0.3921
other	209	1.0	2.60 (1.06, 6.41) 0.0374	1.40 (0.71, 2.77) 0.3341	1.03 (0.53, 1.99) 0.9269
Admission type					
Elective	335	1.0	1.48 (0.48, 4.54) 0.4964	1.06 (0.65, 1.73) 0.8187	0.85 (0.52, 1.40) 0.5330
Emergency/Urgent	2043	1.0	1.33 (1.01, 1.76) 0.0423	0.92 (0.78, 1.08) 0.3156	0.81 (0.68, 0.97) 0.0205
SAPSII					
<43	1151	1.0	1.22 (0.74, 2.01) 0.4393	1.00 (0.76, 1.31) 0.9961	0.62 (0.46, 0.84) 0.0023
≥43	1227	1.0	1.42 (1.04, 1.95) 0.0282	0.90 (0.74, 1.08) 0.2626	0.89 (0.73, 1.09) 0.2504
SOFA					
<7	1173	1.0	1.27 (0.89, 1.82) 0.1822	0.95 (0.75, 1.20) 0.6810	0.68 (0.52, 0.90) 0.0070
≥7	1205	1.0	1.42 (0.96, 2.12) 0.0819	0.91 (0.74, 1.12) 0.3740	0.87 (0.71, 1.08) 0.2160
OASIS					
<38	1161	1.0	1.45 (0.93, 2.26) 0.0988	0.90 (0.71, 1.15) 0.4074	0.63 (0.48, 0.83) 0.0010
≥38	1217	1.0	1.42 (1.01, 1.98) 0.0416	0.94 (0.77, 1.15) 0.5501	0.94 (0.76, 1.16) 0.5799
ARDS					
Mild	493	1.0	1.15 (0.64, 2.07) 0.6363	0.94 (0.64, 1.36) 0.7257	0.74 (0.50, 1.09) 0.1295
Moderate	1110	1.0	1.20 (0.81, 1.78) 0.3596	0.88 (0.70, 1.10) 0.2561	0.75 (0.58, 0.97) 0.0255
Severe	775	1.0	1.39 (0.86, 2.24) 0.1809	0.97 (0.75, 1.25) 0.7933	0.83 (0.62, 1.09) 0.1812

## Discussion

The results of this study suggested that there was an independent association between BMI and the short-term and long-term mortality of ARDS patients according to the Berlin standard. Underweight patients had higher risks of short-term and long-term mortality, while obese patients had lower risks of mortality compared with normal weight patients. In addition, according to the Berlin standard, patients were divided into three groups (mild, moderate, and severe ARDS) based on their PaO2/FiO2 ratio with the first record after ICU admission. Obesity was also found to be associated with a decreased risk of short-term and long-term mortality in ARDS patients despite of the severity.

As far as we know, there were some previous studies about the relationship between BMI and the mortality of ARDS, but their conclusions were controversial (12, 15–20). Most of these studies had shown that BMI was inversely associated with the mortality of the patients with acute lung injury (ALI) or ARDS, and obesity or morbidly obesity could reduce the risk of mortality, which was consistent with our finding (13, 14, 16–20). Other studies suggested that there was no association between BMI and the mortality of ARDS (12, 15). In addition, there were two meta-analyses on BMI and the clinical prognosis of ARDS. Yue et al. reported that in a meta-analysis which included 6,268 patients with ARDS or ALI, the OR of ICU mortality was 0.68 (95% CI 0.57, 0.80) for obese patients and 0.72 (95% CI 0.56, 0.93) for morbidly obese patients respectively (13). Another meta-analysis which included 9 studies showed that obesity significantly decreased the risk of 60-day and 90-day mortality, OR values was 0.84 (95% CI 0.75to –0.94) and 0.38 (95% CI 0.22–0.66) (14). These inconsistent results may be due to the following reasons. First, in different investigations, there were different cut-off values of BMI, different ages, and different races of individuals; Second, the study population was included according to different diagnostic criteria for ARDS and ALI and most of these studies used the American European Consensus Conference definitions; Third, there were significant differences in some studies due to the lack of adjustments for potential important confounding factors, including comorbid illness (such as diabetes mellitus and acute renal insufficiency, which had been shown to be associated with the onset and prognosis of ARDS (16, 21)) and disease severity (APS III score, SIRS score and the value of oxygenation index).

In the present study, we selected patients according to the Berlin definition for ARDS, which is the current definition and guidelines, and we also defined obesity according to WHO standards. We had adjusted for important comorbid illnesses (including Elixhauser Comorbidity index) and disease severity (including SAPSII, OASIS, SOFA, and the value of oxygenation index) because MIMIC-III database had complete patients’ records. In addition, we had investigated the association between BMI and 1-year mortality of ARDS. To our knowledge, this association has not been reported in previous studies.

Our study had a large sample size and adjusted for multiple confounders, which were improvements on previous studies. As a retrospective observational study, we found an association between obesity and a decreased risk of death, however we were unable to establish a causal relationship between obesity and mortality. In fact, ARDS may be over-diagnosed in obese subjects, as the chest wall can more readily induce atelectasis masquerading as bilateral infiltrates on chest x-ray. This leads to (easily reversible) hypoxemia, which makes the subject appear more sick than they really are. On the other hand, due to the existence of collider bias, the real association between obesity and mortality is artificially biased forward being falsely “protective” by conditioning on the diagnosis of ARDS (22, 23). Therefor based on our data, obesity was associated with a decreased risk of mortality for ARDS patients. Due to the collider bias and the possibility that obese patients may be over-diagnosed with ARDS, it was unclear whether obesity paradox existed in these patients.

The physiologic mechanisms behind these associations are not yet clear. We can give some possible reasons why obesity can improve clinical prognosis of ARDS patients. First, obesity is a medical condition with pro-inflammatory components and obese patients have higher cytokines in the peripheral circulation which may induce antioxidant consumption and endothelial damage leading to increased capillary permeability and lung damage (24). However, in animal studies, obesity can lead to impaired neutrophil functions (including cytokines transcription and downstream signaling pathways), the transformation of alveolar macrophages into an anti-inflammatory phenotype, and a reduction in the number of monocytes and adhesion receptors compared with normal weight individuals (25, 26). In addition, previous studies have shown that pro-inflammatory factors in the peripheral circulation of obese ARDS patients are not significantly higher than those of normal weight patients (27). Furthermore, in animal with acute lung injury, it was found that the phenotypes of neutrophils and monocytes/macrophages in BALF and blood of obese rats were changed, making them produce anti-inflammatory and anti-fibrosis effects and preventing further deterioration of lung function (25). Thus the chronic inflammation of obesity may induce tolerance after an acute inflammation, low-grade inflammation may protect the lungs from further damage by preventing more severe secondary inflammation (24). Second, ARDS is a hypercatabolic state. Due to the high lipid bank, obese patients can provide more substrate synthesis energy to meet the higher demands during the initial catabolic phase of the disease (5). Obese patients can also provide more lipoproteins which can bind to endotoxins and reduce their inflammatory effects (4, 21). Third, some studies reported that obesity can reduce the occurrence of ventilator-induced lung injury in ARDS patients (28, 29). In obesity, the altered chest wall dynamic can reduce the impact of airway pressure, thereby providing protection against ventilator-induced lung injury (4). And an animal experiment showed that in obese mice with ARDS, adipose-derived exosomes can protect the lung from endothelial barrier injury and reduce the inflammatory response by inhibiting TRPV4/Ca^2+^ signaling pathway, thus reducing the occurrence of ventilator-related lung injury (30). Finally, clinicians generally consider that there is a higher risk of mortality for obese patients compared with normal weight patients. And this may lead to earlier admission to ICU and prompt prevention measures (including tighter blood sugar control and greater attention to ventilator parameters) which can reduce the risk of death in these patients (31).

Another finding of the present study was that obese patients had higher PEEP, plateau pressure, minute ventilation, and FiO2 than normal weight patients, which have also been described in obese patients in some previous studies. Christian et al. have found that higher PEEP can improve the survival rate of obese ARDS patients (32). In ARDS patients, high PEEP can reduce the lung injury caused by repeated collapse and reopening of the alveoli, thus improving oxygen cooperation. There was a difference in atelectasis between obese and normal patients. In obese patients, respiratory compliance decreased, lung resistance increased, and alveolar atelectasis increased. Therefore, increased PEEP may have a protective effect on obese patients to reduce the damage of repeated collapse and reopening of the alveoli to the lung, thus improving oxygen concentration. In addition, previous studies have found that obese and severely obese ARDS patients need high platform pressure and minute ventilation (24, 33). However, after adjusting for confounding factors, high platform pressure and minute ventilation were not significantly associated with the prognosis of patients. Therefore, we speculated that high PEEP may be another reason why obesity can reduce the risk of mortality in ARDS patients.

In the present study, we had four limitations. First, as a retrospective cohort study, it is impossible to adjust for all confounders. Because several variables were not recorded in MIMIC-III, we lacked information on the clinic risks for ARDS (including sepsis, pneumonia, trauma, and aspiration) which may affect our results. Second, we used the height and weight recorded at ICU admission to calculate BMI. However, it cannot be excluded that whether the patient underwent fluid resuscitation prior to ICU admission. Third, the results presented in this study were associations and do not imply causality, and the molecular and physiological mechanisms behind it need to be further studied and clarified. Fourth, as a single-center study, the results should be interpreted with caution when referring to other populations and regions.

## Conclusion

In conclusion, our investigation indicates that BMI was independently associated with short-term and long-term mortality of ARDS patients according to the Berlin standard. Compared with normal weight, obesity had lower risk of mortality for patients with ARDS. The relationship needs to be further validated in other populations, and the mechanisms behind these associations need to be further investigated. Understanding how obesity decreased the risk of mortality in ARDS may affect the treatment strategies and clinical outcomes for many patients.

## Data Availability Statement

The original contributions presented in the study are included in the article/**Supplementary Material**. Further inquiries can be directed to the corresponding author.

## Author Contributions

WZ designed the study, collected, and analyzed data, and contributed to writing this manuscript. YW collected and analyzed data. JW and WL designed and supervised the study and drafted the manuscript. All authors contributed to the article and approved the submitted version.

## Funding

This study was funded by General Projects of Social Development in Shaanxi Province (2019SF-151) and Key Research and Development Project of Shaanxi Province (S2020-YF-ZDCXL-ZDLSF-0048).

## Conflict of Interest

YW was employed by the company Ruibiao (Wuhan) Biotechnology Co.

The remaining authors declare that the research was conducted in the absence of any commercial or financial relationships that could be construed as a potential conflict of interest.
