# Supplementary material for: Association Between Obesity and Short-And Long-Term Mortality in Patients With Acute Respiratory Distress Syndrome Based on the Berlin Definition
Source: Front Endocrinol (Lausanne). 2021 Feb 12;11:611435. doi: 10.3389/fendo.2020.611435 (PMC7907504; doi:10.3389/fendo.2020.611435)
Supplement: Supplementary file 1 [file Table_1.docx]

**Favorable short-and long-term mortality in obesity patients with acute respiratory distress syndrome based on the Berlin Definition**

**Table S1 Univariate logistics regression analysis of all patients on ICU and in-hospital mortality**

| Variables | ICU mortality  OR 95% CI p-value | In-hospital mortality  OR 95% CI p-value |
| --- | --- | --- |
| Age (years) | 1.02 (1.01, 1.02) <0.0001 | 1.02 (1.01, 1.02) <0.0001 |
| Gender | | |
| Male | 1.0 | 1.0 |
| Female | 1.09 (0.90, 1.33) 0.3642 | 1.05 (0.88, 1.26) 0.5823 |
| Ethnicity | | |
| Other | 1.0 | 1.0 |
| Black | 1.51 (1.05, 2.17) 0.0260 | 1.51 (1.06, 2.13) 0.0213 |
| Caucasian | 0.76 (0.54, 1.06) 0.1036 | 0.82 (0.59, 1.12) 0.2104 |
| BMI | | |
| Normal weight | 1.0 | 1.0 |
| Underweight | 1.66 (1.29, 2.14) <0.0001 | 1.61 (1.27, 2.05) <0.0001 |
| Overweight | 0.87 (0.75, 1.01) 0.0765 | 0.87 (0.75, 0.99) 0.0390 |
| Obesity | 0.63 (0.53, 0.73) <0.0001 | 0.62 (0.54, 0.71) <0.0001 |
| ICU type | | |
| CCU/CSRU | 1.0 | 1.0 |
| MICU | 2.19 (1.37, 3.51) 0.0011 | 2.46 (1.57, 3.86) <0.0001 |
| SICU/TSICU | 4.09 (2.59, 6.44) <0.0001 | 4.89 (3.16, 7.58) <0.0001 |
| Admission type |  |  |
| Elective | 1.0 | 1.0 |
| Emergency/Urgent | 1.47 (1.09, 1.98) 0.0111 | 1.62 (1.22, 2.15) 0.0009 |
| Vital signs within 24h after ICU admission | | |
| SPO2 | 0.89 (0.87, 0.92) <0.0001 | 0.92 (0.89, 0.94) <0.0001 |
| Heart rate (bpm) | 1.01 (1.01, 1.02) 0.0002 | 1.01 (1.00, 1.01) 0.0301 |
| Temperature(℃) | 0.65 (0.57, 0.74) <0.0001 | 0.60 (0.53, 0.68) <0.0001 |
| MAP(mmHg) | 0.96 (0.95, 0.97) <0.0001 | 0.96 (0.95, 0.97) <0.0001 |
| Laboratory data on the first day after ICU admission | | |
| Arterial pH | 0.01 (0.00, 0.02) <0.0001 | 0.02 (0.01, 0.06) <0.0001 |
| PaCO2 (mm Hg) | 1.00 (0.99, 1.01) 0.5891 | 1.00 (0.99, 1.01) 0.7480 |
| [Lactic](C:/Users/Administrator/AppData/Local/youdao/dict/Application/8.9.5.0/resultui/html/index.html#/javascript:;) [acid](C:/Users/Administrator/AppData/Local/youdao/dict/Application/8.9.5.0/resultui/html/index.html#/javascript:;) | 1.28 (1.22, 1.33) <0.0001 | 1.23 (1.18, 1.28) <0.0001 |
| PaO2/FiO2 (mmHg) | 1.00 (0.99, 1.00) <0.0001 | 1.00 (1.00, 1.00) 0.0002 |
| Renal replacement therapy |  |  |
| No | 1.0 | 1.0 |
| Yes | 1.34 (0.90, 1.99) 0.1448 | 1.52 (1.05, 2.20) 0.0277 |
| Vasopressor |  |  |
| No | 1.0 |  |
| Yes | 1.14 (0.94, 1.37) 0.1869 | 1.01 (0.85, 1.21) 0.8853 |
| Metastatic cancer |  |  |
| No | 1.0 | 1.0 |
| Yes | 2.38 (1.70, 3.32) <0.0001 | 2.29 (1.65, 3.19) <0.0001 |
| Liver disease |  |  |
| No | 1.0 | 1.0 |
| Yes | 1.45 (1.09, 1.93) 0.0117 | 1.66 (1.26, 2.17) 0.0003 |
| Renal failure |  |  |
| No | 1.0 | 1.0 |
| Yes | 1.30 (1.00, 1.68) 0.0490 | 1.57 (1.24, 2.00) 0.0002 |
| Diabetes |  |  |
| No | 1.0 | 1.0 |
| Yes | 0.89 (0.72, 1.11) 0.3030 | 0.90 (0.73, 1.10) 0.3094 |
| Chronic pulmonary disease |  |  |
| No | 1.0 | 1.0 |
| Yes | 0.99 (0.80, 1.23) 0.9213 | 1.05 (0.86, 1.29) 0.6207 |
| Cerebrovascular disease |  |  |
| No | 1.0 | 1.0 |
| Yes | 0.66 (0.48, 0.92) 0.0129 | 0.79 (0.59, 1.06) 0.1149 |
| Chronic heart disease |  |  |
| No | 1.0 | 1.0 |
| Yes | 1.37 (1.13, 1.66) 0.0014 | 1.58 (1.32, 1.90) <0.0001 |
| Sepsis |  |  |
| No | 1.0 | 1.0 |
| Yes | 1.22 (1.01, 1.48) 0.0417 | 1.29 (1.08, 1.55) 0.0061 |
| Elixhauser comorbidity score | 1.05 (1.03, 1.06) <0.0001 | 1.06 (1.05, 1.07) <0.0001 |
| Severity of organ dysfunction | | |
| SAPSII | 1.05 (1.04, 1.05) <0.0001 | 1.05 (1.04, 1.05) <0.0001 |
| OASIS | 1.05 (1.04, 1.07) <0.0001 | 1.05 (1.04, 1.06) <0.0001 |
| SOFA | 1.15 (1.12, 1.18) <0.0001 | 1.13 (1.11, 1.16) <0.0001 |
| Characteristics of mechanical ventilation on the first day after ICU admission | | |
| Tidal volume (ml/kg PBW) | 0.99 (0.96, 1.02) 0.4042 | 0.98 (0.96, 1.01) 0.2468 |
| PEEP (cmH2O) | 1.04 (1.01, 1.07) 0.0031 | 1.02 (0.99, 1.05) 0.1468 |
| Plateau pressure (cmH2O) | 1.05 (1.03, 1.07) <0.0001 | 1.04 (1.02, 1.05) <0.0001 |
| Minute ventilation (l/min) | 1.02 (1.00, 1.03) 0.0580 | 1.01 (0.99, 1.02) 0.3034 |
| FiO2 (%) | 1.02 (1.01, 1.03) <0.0001 | 1.01 (1.00, 1.02) 0.0026 |

BMI: Body Mass Index; ICU: intensive care units; CCU: coronary care unit; CSRU: cardiac surgery recovery unit; MICU: medical intensive care unit; SICU: surgical intensive care unit; TSICU: thoracic surgery Intensive care unit; MAP: Mean Arterial Pressure; PaO2/FiO2: arterial oxygen partial pressure/ fraction of inspired oxygen; SAPSII: Simplified Acute Physiology Score ; OASIS: Oxford Acute Severity of Illness Score; SOFA:Sequential Organ Failure Assessment; PBW: predicted body weight ; PEEP: positive end-expiratory pressure; bpm: breaths per minute;

**Table S2 Univariate Cox regression analysis of all patients on 1-year mortality**

| Variables | 1-year mortality  HR 95% CI p-value |
| --- | --- |
| Age (years) | 1.03 (1.02, 1.03) <0.0001 |
| Gender | |
| Male | 1.0 |
| Female | 1.04 (0.92, 1.17) 0.5625 |
| Ethnicity | |
| Other | 1.0 |
| Black | 1.52 (1.19, 1.95) 0.0008 |
| Caucasian | 1.02 (0.81, 1.29) 0.8385 |
| BMI | |
| Normal weight | 1.0 |
| Underweight | 1.66 (1.29, 2.14) <0.0001 |
| Overweight | 0.87 (0.75, 1.01) 0.0765 |
| Obesity | 0.63 (0.53, 0.73) <0.0001 |
| ICU type | |
| CCU/CSRU | 1.0 |
| MICU | 1.79 (1.34, 2.39) <0.0001 |
| SICU/TSICU | 2.94 (2.23, 3.88) <0.0001 |
| Admission type | |
| Elective | 1.0 |
| Emergency/Urgent | 1.38 (1.14, 1.67) 0.0009 |
| Vital signs within 24h after ICU admission | |
| SPO2 | 0.95 (0.94, 0.97) <0.0001 |
| Heart rate (bpm) | 1.00 (1.00, 1.00) 0.8307 |
| Temperature(℃) | 0.67 (0.62, 0.72) <0.0001 |
| MAP(mmHg) | 0.97 (0.96, 0.98) <0.0001 |
| Laboratory data on the first day after ICU admission | |
| Arterial pH | 0.08 (0.03, 0.19) <0.0001 |
| PaCO2 (mm Hg) | 1.00 (1.00, 1.01) 0.3111 |
| [lactic](C:/Users/Administrator/AppData/Local/youdao/dict/Application/8.9.5.0/resultui/html/index.html#/javascript:;) [acid](C:/Users/Administrator/AppData/Local/youdao/dict/Application/8.9.5.0/resultui/html/index.html#/javascript:;) | 1.13 (1.11, 1.16) <0.0001 |
| PaO2/FiO2 (mmHg) | 1.00 (1.00, 1.00) 0.0009 |
| Renal replacement therapy |  |
| No | 1.0 |
| Yes | 1.39 (1.09, 1.76) 0.0073 |
| Vasopressor |  |
| No | 1.0 |
| Yes | 1.00 (0.89, 1.13) 0.9636 |
| Metastatic cancer |  |
| No | 1.0 |
| Yes | 2.51 (2.07, 3.04) <0.0001 |
| Liver disease |  |
| No | 1.0 |
| Yes | 1.31 (1.09, 1.57) 0.0039 |
| Renal failure |  |
| No | 1.0 |
| Yes | 1.61 (1.38, 1.87) <0.0001 |
| Diabetes |  |
| No | 1.0 |
| Yes | 0.91 (0.79, 1.05) 0.1960 |
| Chronic pulmonary disease |  |
| No | 1.0 |
| Yes | 1.23 (1.08, 1.40) 0.0025 |
| Cerebrovascular disease |  |
| No | 1.0 |
| Yes | 0.86 (0.71, 1.05) 0.1469 |
| Chronic heart disease |  |
| No | 1.0 |
| Yes | 1.64 (1.46, 1.86) <0.0001 |
| Sepsis |  |
| No | 1.0 |
| Yes | 1.23 (1.08, 1.39) 0.0013 |
| Elixhauser comorbidity score | 1.05 (1.04, 1.06) <0.0001 |
| SAPSII | 1.04 (1.03, 1.04) <0.0001 |
| OASIS | 1.04 (1.03, 1.05) <0.0001 |
| SOFA | 1.09 (1.07, 1.11) <0.0001 |
| Characteristics of mechanical ventilation on the first day after ICU admission | |
| Tidal volume (ml/kg PBW) | 1.00 (0.98, 1.01) 0.7309 |
| PEEP (cmH2O) | 1.00 (0.98, 1.02) 0.7587 |
| Plateau pressure (cmH2O) | 1.02 (1.01, 1.03) 0.0021 |
| Minute ventilation (l/min) | 1.00 (0.99, 1.01) 0.7999 |
| FiO2 (%) | 1.01 (1.00, 1.01) 0.0146 |

BMI: Body Mass Index; ICU: intensive care units; CCU: coronary care unit; CSRU: cardiac surgery recovery unit; MICU: medical intensive care unit; SICU: surgical intensive care unit; TSICU: thoracic surgery Intensive care unit; MAP: Mean Arterial Pressure; PaO2/FiO2: arterial oxygen partial pressure/ fraction of inspired oxygen; SAPSII: Simplified Acute Physiology Score ; OASIS: Oxford Acute Severity of Illness Score; SOFA:Sequential Organ Failure Assessment; PBW: predicted body weight ; PEEP: positive end-expiratory pressure; bpm: breaths per minute;
